# Two new species of *Coecobrya* (Collembola, Entomobryidae) from China, with an updated key to the Chinese species of the genus

**DOI:** 10.3897/zookeys.498.9491

**Published:** 2015-04-21

**Authors:** Guo-Liang Xu, Feng Zhang

**Affiliations:** 1 School of Geographical Sciences, Guangzhou University, Guangzhou 510006, P. R. China; 2 Department of Entomology, College of Plant Protection, Nanjing Agricultural University, Nanjing 210095, P. R. China

**Keywords:** *Coecobrya sanmingensis* sp. n., *Coecobrya qinae* sp. n., chaetotaxy

## Abstract

Two new *Coecobrya* species, which were newly collected in 2014, are described from China. *Coecobrya sanmingensis* **sp. n.** from southeast China (Fujian) is the fourth 1+1 eyed species in the genus; it can be distinguished from other three species by the ciliate chaetae X and X_2-4_ on the ventral side of head, the abundant chaetae on the trochanteral organ, a large outer tooth on the unguiculus, the absence of smooth manubrial chaetae, and the dorsal chaetotaxy. *Coecobrya qinae* **sp. n.** from southwest China (Yunnan) is characterized by paddle-like S-chaetae of Ant. III organ, ciliate chaetae X, X_2_ and X_4_ posterior to labium, medial macrochaetae on the mesothorax, and 5+5 central and 2+2 lateral macrochaetae on the fourth abdominal segment. An updated key to the Chinese species of *Coecobrya* is given.

## Introduction

Deharveng (1990), Chen and Christiansen (1997) and Zhang et al. (2009, 2011a) made great contributions to the modern taxonomy of the genus *Coecobrya*. Its members have plurichaetotic chaetotaxy, no labral papillae, an inverted intrusion on the labral margin which is U-shaped, labial chaetae MELL always smooth, reduced eye number (0 to 3 eyes per side), pigment reduced or absent, antennal apical bulb absent, falcate mucro with a basal spine, tenaculum with 4+4 teeth and one large striate chaeta, and scales and dental spines absent (Zhang et al. 2009). The genus is worldwide and very abundant in Southeast and East Asia. So far, about approximately one quarter (12/47) of species have been reported from China. Five of them were discovered during recent expeditions in this country (Xu et al. 2012; Zhang and Dong 2014). Here, two new species, collected in 2014, are described from southern China. An updated key to the Chinese species of *Coecobrya* is also given.

## Materials and methods

Specimens were cleared in Nesbitt’s fluid, mounted under a coverslip in Marc André II solution, and studied using a Nikon E80i microscope. Photographs were enhanced with Photoshop CS5. The labial chaetae terminology follows Gisin’s system (1967). The dorsal and ventral chaetotaxy of head and the Ant. III organ are described after Chen and Christiansen (1993). Dorsal body chaetae are designated following Szeptycki (1979) and Zhang et al. (2011b). The number of macrochaetae is given by half-tergite in the descriptions (left side of tergites drawn in figures). Tergal S-chaetotaxic formula follows Zhang and Deharveng (2014). Type material is deposited in the collections of the Department of Entomology, College of Plant Protection, Nanjing Agricultural University (NJAU), P. R. China.

Abbreviations: Th. – thoracic segment; Abd. – abdominal segment; Ant. – antennal segment; mac – macrochaeta/ae; mic – microchaeta/ae; ms – S-microchaeta/ae; sens – ordinary tergal S-chaeta/ae.

## Taxonomy

### Coecobrya sanmingensis sp. n.

Taxon classificationAnimaliaCollembolaEntomobryidae

http://zoobank.org/5241A8F2-F00E-4533-939B-8C79D22C3582

Figs 1 , 3–13 , 14–18 , Table 1

#### Type locality.

China, Fujian, Sanming, 26.500°N, 117.717°E, altitude 707 m.

#### Material.

Holotype: ♀ on slide, China, Fujian Province, Sanming City, Guanzhuang National Forestry Farm, 26.500°N, 117.717°E, altitude 707 m, 17 September 2014, Daoyuan YU leg. (# Sanming 9-2). Paratypes: 1 ♂, 1 ♀, and 1 juvenile of unclear sex on slides and 5 juveniles in alcohol, same data as holotype.

#### Description.

**Figures 1–2.**
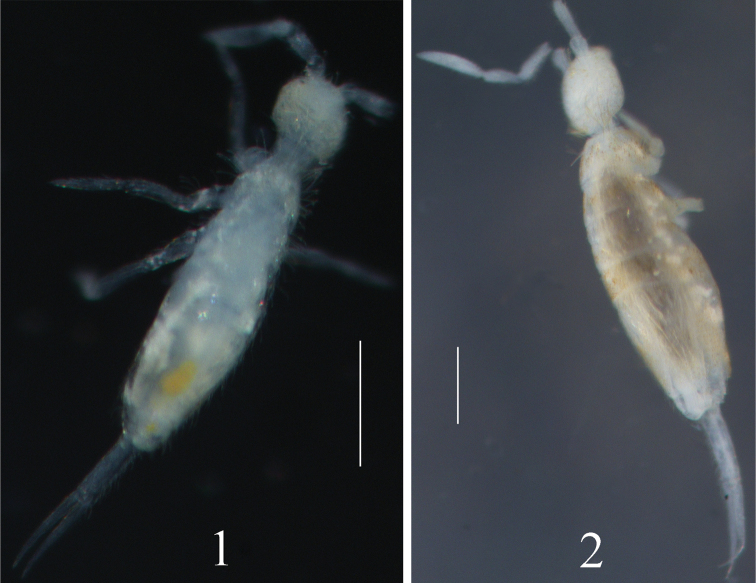
Habitus. **1** *Coecobrya sanmingensis* sp. n. **2** *Coecobrya qinae* sp. n. Scale bars: 300 μm.

Body length up to 1.09 mm. Body pale (Fig. 1).

**Figures 3–13.**
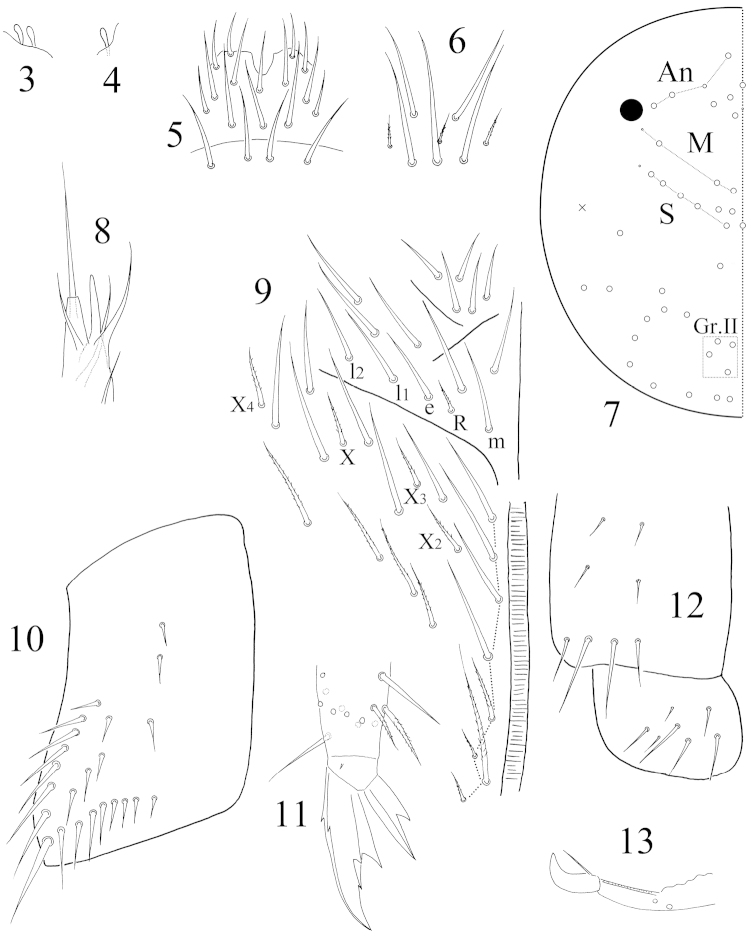
*Coecobrya sanmingensis* sp. n. **3** Ant. III organ **4** Ant. IV subapical organ **5** labrum **6** clypeal chaetae **7** dorsal cephalic chaetotaxy **8** lateral process of labial palp E **9** chaetae on the ventral side of head **10** trochanteral organ **11** hind claw **12** ventral face and lateral flap of ventral tube **13** mucro.

Antenna 1.96‒2.02X as long as cephalic diagonal. Antennal segment ratio as I: II: III: IV = 1: 1.69: 0.88‒1.23: 1.94‒3.08. Smooth spiny mic at base of antennae 3 dorsal, 3 ventral on Ant. I, 1 internal, 1 external and 1 ventral on Ant II. Two internal S-chaetae of Ant III organ expanded (Fig. 3). Ant. IV subapical organ thin, distally expanded (Fig. 4). Long smooth straight chaetae absent.

Eyes 1+1. Prelabral and labral chaetae 4/ 5, 5, 4, all smooth; median three chaetae of the first row longer than lateral ones (Fig. 5). Eight clypeal chaetae arranged in three rows; median three ciliate and much smaller (Fig. 6). Dorsal cephalic chaetotaxy with 4 antennal (An), 3 median (M) and 5 sutural (S) mac; Gr. II with 4 mac (Fig. 7). Mandibles 4+5 teeth. Subapical chaeta of maxillary outer lobe larger than apical one; 3 smooth sublobal hairs on maxillary outer lobe (Fig. 22). Papillae A‒E of labial palp with 0, 5, 0, 4, 4 guard chaetae respectively; lateral process of papillae E slightly thicker than normal chaetae, with tip beyond apex of labial papilla (Fig. 8). Labial chaetae as mRel_1_l_2_, R ciliate, R/m=0.4; chaetae X and X_2‒4_ ciliate. Cephalic groove with 8(7) chaetae, anterior four smooth and posterior ones ciliate (Fig. 9).

Trochanteral organ with 23 smooth spiny chaetae; 16 in arms and 7 between them (Fig. 10). Partial inner differentiated tibiotarsal chaetae “smooth” with ciliations closely appressed to axis. Tibiotarsi most distally with 11 chaetae in a whorl. Unguis with 3 inner teeth; 2 paired teeth unequal, outer one larger. Unguiculus with a large outer tooth. All tenent hairs pointed (Fig. 11). Abd. IV 3.56‒3.83X as long as Abd. III along dorsal midline. Ventral tube anteriorly with 3+3 large ciliate chaetae; posteriorly with 4 distal and 4‒5 proximal smooth chaetae; each lateral flap with 7 smooth chaetae (Fig. 12). Manubrium without smooth chaetae. Manubrial plaque with 2+2 pseudopores and 3+3 ciliate chaetae. Distal smooth part of dens 1.0X as long as mucro. Mucro falcate (Fig. 13).

**Figures 14–18.**
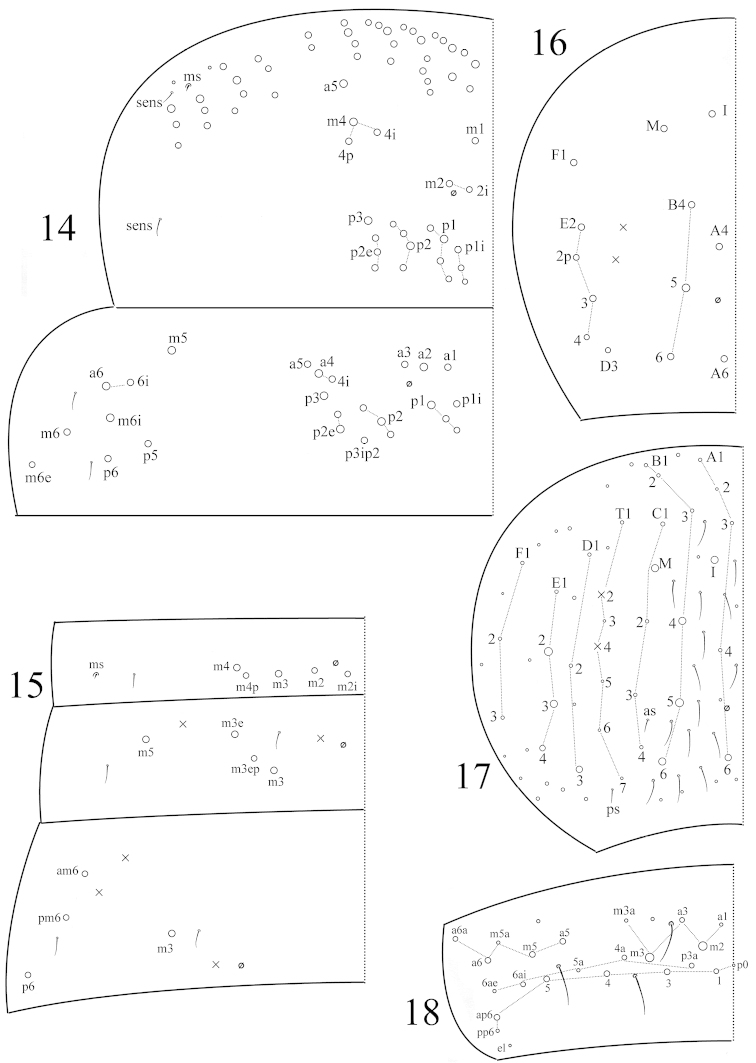
Tergal chaetotaxy in *Coecobrya sanmingensis* sp. n. **14** thorax **15** Abd. I‒III **16** Abd. IV of adults **17** Abd. IV of juveniles (possibly 2^nd^ instar) **18** Abd. V of juveniles.

Th. II with 3 (m1, m2, m2i) medio-medial, 3 (m4, m4i, m4p) medio-lateral, about 15 posterior mac, 1 ms and 2 sens. Th. III with 25 mac and 2 lateral sens; mac m5i absent; p5 and m6i as mac (Fig. 14). Abd. I with 5 (m2–4, m2i, m4p) mac, 1 ms and 1 sens; sens inner to ms. Abd. II with 3 (m3, m3e, m3ep) central, 1 (m5) lateral mac and 2 sens. Abd. III with 1 (m3) central, 3 (am6, pm6, p6) lateral mac, and 2 sens; ms absent (Fig. 15). Abd. IV with 7 (I, M, A4, A6, B4‒6) central, 6 lateral mac (D3, E2–4, E2p, F1) (Fig. 16) and 17 sens; two lateral sens (as, ps) of normal length and others elongate (Fig. 17). Abd. V with 3 sens (Fig. 18).

**Figures 19–26.**
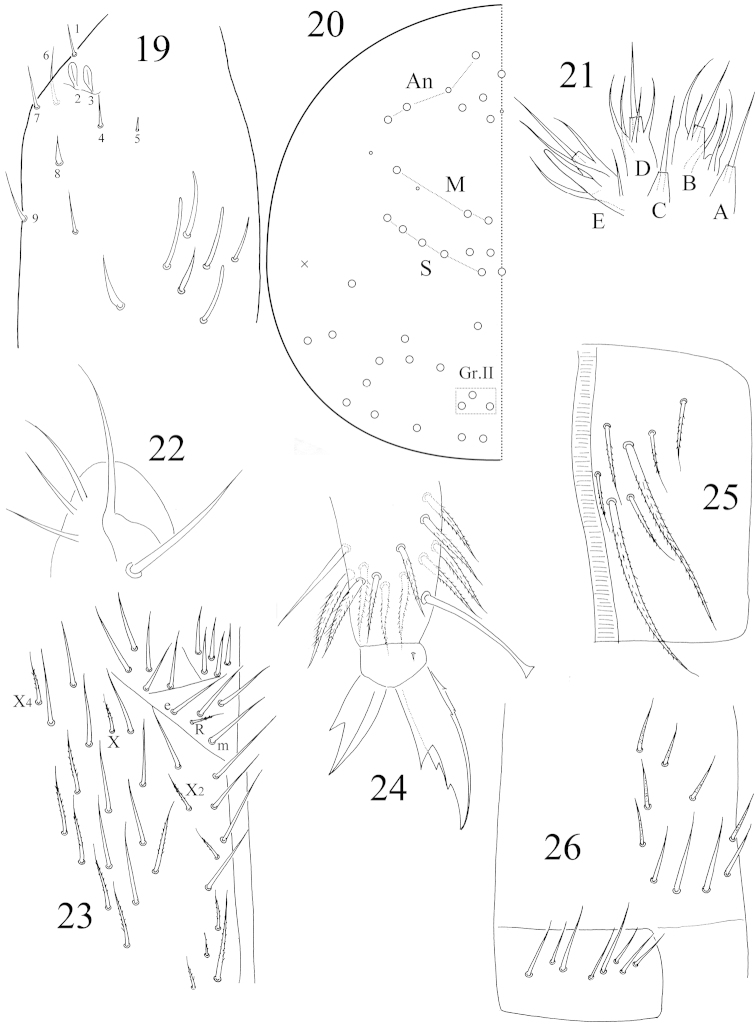
*Coecobrya qinae* sp. n. **19** Ant. III organ **20** dorsal cephalic chaetotaxy **21** labial palp **22** maxillary outer lobe, same as *Coecobrya sanmingensis* sp. n. **23** chaetae on the ventral side of head **24** hind claw **25** anterior face of ventral tube **26** posterior face and lateral flap of ventral tube.

#### Etymology.

Named after the type locality.

#### Ecology.

In soil.

#### Remarks.

*Coecobrya sanmingensis* sp. n. is the fourth 1+1 eyed species of the genus. These eyed species share many features such as the presence of eyes, tip of lateral process of labial palp E beyond the same papillae, dorsal cephalic chaetotaxy (4An, 3M, 5S), partial inner differentiated tibiotarsal chaetae “smooth”, 2+2 pseudopores on the manubrial plaque, 3+3 medio-medial mac on Th. II, 1+1 central and 3+3 lateral mac on Abd. III, and 6 lateral mac on Abd. IV. They can be separated by antennal long smooth chaetae, chaetae on the ventral side of head, trochanteral organ, unguiculus outer edge, smooth manubrial chaetae, and dorsal chaetotaxy (Table 1).

**Table 1.** Comparison among the four 1+1 eyed *Coecobrya* species. (?, unknown; +, present; -, absent).

Characters	*sanmingensis* sp. n.	*boneti*	*indonesiensis*	*tukmeas*
Antennal long smooth straight chaetae	-	+	?	+
Gr. II on dorsal head	4	4	5	4
Labial chaeta R	short, ciliate	tiny, smooth	long, smooth	tiny, smooth
Chaetae posterior to labium X X_2_ X_3_ X_4_	ciliate ciliate ciliate ciliate	smooth tiny - tiny	smooth - - Smooth	smooth - - tiny
Trochanteral organ	23	11‒15	9	11‒14
Large outer tooth on unguiculus	+	-	-	-
Chaetae of ventral tube anterior face posterior face lateral flap	? 8‒9 7	6 8 9	10 ? 9‒10	5‒6 6 8
Manubrial smooth chaetae	-	+	+	+
Ciliate chaetae on manubrial plaque	3+3	3+3	3+3	2+2
Chaetotaxy of Th. II medio-lateral (m4+) Gr. VI (p4+) p5	3 0 0	3 2 1	2 2 0	2 2 0
Mac m5i on Th. III	-	-	-	+
Chaetotaxy of Abd. I	5+5	6+6	6+6	4+4
Chaetotaxy of Abd. II inner to arch M-arch	3 0 3	4 1 3	4(5) 1(2) 3	3 1 2
ms on Abd. III	-	+	?	-
Central mac on Abd. IV	7	6	7	6

### Coecobrya qinae sp. n.

Taxon classificationAnimaliaCollembolaEntomobryidae

http://zoobank.org/A5C29F1B-B569-4BF7-B236-5BBF960ECAE1

Figs 2 , 19–26 , 27–30

#### Type locality.

China, Yunnan, 26.643°N, 98.905°E, altitude 1149 m.

#### Material.

Holotype: ♀ on slide, China, Yunnan Province, 228 Provincial Highway, 26.643°N, 98.905°E, altitude 1149 m, 11 October 2014, Chunyan QIN leg. (# 14YN2). Paratype: ♀ on slide, same data as holotype.

#### Description.

Body length up to 1.49 mm. Body with light orange pigment (Fig. 2).

Antenna 1.80X as long as cephalic diagonal. Antennal segment ratio as I: II: III: IV = 1: 2.06: 2.00: 3.06. Smooth spiny mic at base of antennae 3 dorsal, 3 ventral on Ant. I, 1 internal, 1 external and 1 ventral on Ant. II. Ant. II distally with 1 expanded S-chaeta. Two internal S-chaetae of Ant. III organ paddle-like, expanded; chaeta 8 dagger-like (Fig. 19). Long smooth straight chaetae absent.

Eyes absent. Prelabral and labral chaetae 4/ 5, 5, 4, all smooth. Clypeal chaetae not clearly seen. Dorsal cephalic chaetotaxy with 4 antennal (An), 3 median (M) and 5 sutural (S) mac; Gr. II with 3 mac (Fig. 20). Mandibles 4+5 teeth. Papillae A‒E of labial palp with 0, 5, 0, 4, 4 guard chaetae respectively; lateral process of papillae E thicker than normal chaetae, with tip beyond apex of labial papilla E (Fig. 21). Subapical chaeta of maxillary outer lobe slightly larger than apical one; 3 smooth sublobal hairs on maxillary outer lobe (Fig. 22). Labial chaetae as mRel_1_l_2_, R ciliate, R/m=0.5; chaetae X, X_2_ and X_4_ ciliate. Cephalic groove with 8 chaetae, four of them smooth and others ciliate (Fig. 23).

Trochanteral organ not clearly seen. Partial inner differentiated tibiotarsal chaetae ciliate with ciliations not closely appressed to axis. Tibiotarsi most distally with 11 chaetae in a whorl. Unguis with 3 inner teeth; 2 paired teeth unequal, outer one larger. Unguiculus with a large outer tooth. All tenent hairs clavate (Fig. 24). Abd. IV 3.65X as long as Abd. III along dorsal midline. Ventral tube anteriorly with 7+7 ciliate chaetae; two of them much larger than others (Fig. 25); posteriorly with 4 distal smooth and 6 proximal weakly ciliate chaetae; each lateral flap with 8 smooth chaetae (Fig. 26). Manubrium without smooth chaetae. Manubrial plaque with 2+2 pseudopores and 2+2 ciliate chaetae. Distal smooth part of dens 1.2X as long as mucro. Mucro falcate.

**Figures 27–30.**
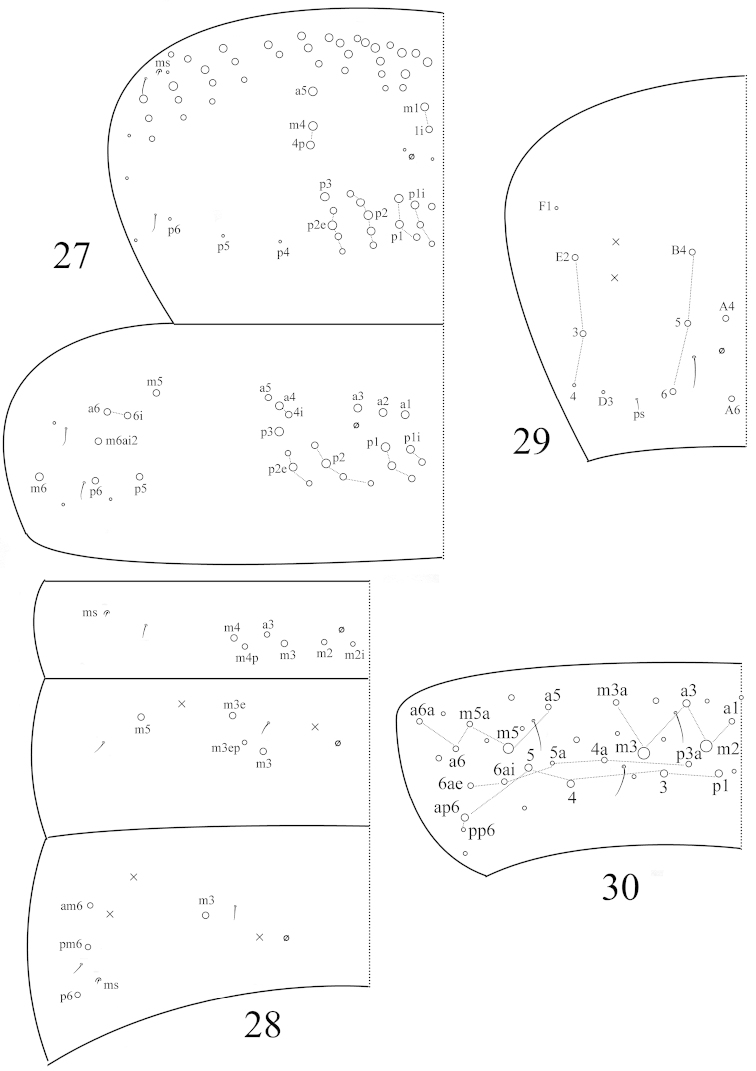
Tergal chaetotaxy in *Coecobrya qinae* sp. n. **27** thorax **28** Abd. I‒III **29** Abd. IV **30** Abd. V.

Th. II with 2(1) (m1, m1i) medio-medial, 2 (m4, m4p) medio-lateral, 16‒17 posterior mac, 1 ms and 2 sens; mac m1i sometimes absent; mac m2 and p4‒6 as mic. Th. III with 24‒26 mac and 2 lateral sens; mac m5i absent; p5 and m6ai2 as mac (Fig. 27). Abd. I with 6 (a3, m2–4, m2i, m4p) mac, 1 ms and 1 sens; sens inner to ms. Abd. II with 3 (m3, m3e, m3ep) central, 1 (m5) lateral mac and 2 sens. Abd. III with 1 (m3) central, 3 (am6, pm6, p6) lateral mac, 1 ms and 2 sens (Fig. 28). Abd. IV with 5 central (A4, A6, B4‒6) and 2 lateral mac (E2–3); number of sens not clearly seen (Fig. 29). Abd. V with 3 sens (Fig. 30).

#### Etymology.

Named after the collector of the specimens.

#### Ecology.

Among the decompositing leaf litter.

#### Remarks.

*Coecobrya qinae* sp. n. is characterized by paddle-like S-chaetae of Ant. III organ, ciliate chaetae X, X_2_ and X_4_ posterior to labium, medial mac on Th. II, and 5+5 (mac I, M absent) central and 2+2 lateral mac on Abd. IV. It is most similar to *Coecobrya tropicalis* Qu, Chen & Greenslade, 2007 in absence of eyes, smooth manubrial chaetae absent, inner differentiated tibiotarsal chaetae, and chaetotaxy of Abd. I‒III. It differs from it in paddle-like S-chaetae on Ant. III organ, thicker lateral process of labial palp, presence of ciliate chaetae X, X_2_ and X_4_ posterior to labium, 2+2 ciliate chaetae on manubrial plaque, absence of mac m2, m2i and p4 on Th. II, and unusual arrangement of central mac on Abd. IV.

### Key to the Chinese species of *Coecobrya*

**Table:** 

1	Eyes present	**2**
–	Eyes absent	**4**
2	Eyes 1+1	***sanmingensis* sp. n.**
–	Eyes 3+3	**3**
3	Body violet-bluish; unguiculus outer edge smooth	***mulun* Zhang, Qu & Deharveng, 2010**
–	Body whitish; unguiculus outer edge with a large tooth	***qin* Zhang & Dong, 2014**
4	Manubrium with dorsal smooth chaetae	**5**
–	Manubrium without dorsal smooth chaetae	**8**
5	Tibiotarsus with rows of “smooth” differentiated chaetae	**6**
–	Tibiotarsus without rows of “smooth” differentiated chaetae	**7**
6	Abd. I with 6+6 central mac; Th. II with 3+3 medio-medial mac	***tenebricosa* (Folsom, 1902)**
–	Abd. I with 4(3)+4(3) central mac; Th. II with 1+1 medio-medial mac	***brevis* Xu, Yu & Zhang, 2012**
7	Abd. IV with 3+3 central and 4+4 lateral mac	***oligoseta* Chen & Christiansen, 1997**
–	Abd. IV with 4+4 central and 3+3 lateral mac	***pani* Xu, Yu & Zhang, 2012**
8	Abd. III with 1+1 central mac	**9**
–	Abd. III with 2+2 central mac	**11**
9	Unguiculus truncate; Abd. IV with 4+4 central mac	***draconis* Zhang & Dong, 2014**
–	Unguiculus acuminate; Abd. IV with more than 4+4 central mac	**10**
10	Abd. IV with 7+7 central and 6+6 lateral mac	***communis* Chen & Christiansen, 1997**
–	Abd. IV with 5+5 central and 2+2 lateral mac	***qinae* sp. n.**
11	Abd. IV with 5+5 central mac	**12**
–	Abd. IV with at least 7+7 central mac	**13**
12	Dorsal head with 5+5 sutural mac; Th. II with 3+3 medio-lateral mac	***huangi* Chen & Christiansen, 1997**
–	Dorsal head with 3+3 sutural mac; Th. II with 2+2 medio-lateral mac	***xui* Zhang & Dong, 2014**
13	Abd. IV with 7+7 central mac	***liui* Wang, Chen & Christiansen, 2002**
–	Abd. IV with at least 8+8 central mac	***tibetensis* Chen & Christiansen, 1997**

**Table 2.** Comparison between *Coecobrya qinae* sp. n. and *Coecobrya tropicalis*.

Characters	*Coecobrya qinae* sp. n.	*Coecobrya tropicalis* Qu et al., 2007
Ant. III organ	paddle-like	rod-like
lateral process of labial palp	thick	thin
Chaetae posterior to labium X X_2_ X_4_	ciliate ciliate ciliate	smooth mic absent smooth mic
Chaetae on manubrial plaque	2+2	3+3
Chaetotaxy of Th. II m1+ m2+	2 2 0	3(2) 1 2
Inner mac on Abd. II	3	2(3)
Chaetotaxy of And. IV I M B4 Lateral mac	5+5 absent absent present 2+2	5+5 present present absent 4+4

## Supplementary Material

XML Treatment for Coecobrya sanmingensis

XML Treatment for Coecobrya qinae
